# circDENND1B Participates in the Antiatherosclerotic Effect of IL-1β Monoclonal Antibody in Mouse by Promoting Cholesterol Efflux via miR-17-5p/Abca1 Axis

**DOI:** 10.3389/fcell.2021.652032

**Published:** 2021-04-29

**Authors:** Fei Xu, Li Shen, Han Chen, Rui Wang, Tongtong Zang, Juying Qian, Junbo Ge

**Affiliations:** ^1^Department of Cardiology, Zhongshan Hospital, Fudan University, Research Unit of Cardiovascular Techniques and Devices, Chinese Academy of Medical Sciences, Shanghai, China; ^2^National Clinical Research Center for Interventional Medicine, Shanghai, China

**Keywords:** atherosclerosis, circular RNA, inflammation, IL-1β mAb, cholesterol efflux

## Abstract

Inflammation is a crucial mediator of atherosclerosis, and several therapeutic methods that focus on inflammatory cytokines, including interleukin-1β (IL-1β), have proven effective in preventing atherogenesis. Circular RNAs (circRNAs) are a subclass of non-coding RNAs (ncRNAs) that can exert critical functions in the regulation of atherosclerosis. Here, using circRNA sequencing, we revealed that circRNA circDENND1B (mmu_circ_0000081) is a promising novel mediator of atherosclerosis in mouse. The expression of circDENND1B is negatively related to the progression of atherosclerosis and foam cell formation, and the upregulation of circDENND1B significantly alleviates foam cell formation induced by ox-LDL by promoting cholesterol efflux. Moreover, circDENND1B participates in the anti-atherosclerotic effect of IL-1β monoclonal antibody (IL-1β mAb), both *in vivo* and *in vitro*. With bioinformatic prediction and RNA pull-down assays, we determined that circDENND1B sponges mmu-miR-17-5p to promote *Abca1* expression in cells treated with IL-1β mAb. Our study revealed that circDENND1B, a novel regulator of cholesterol efflux, is a potential therapeutic target in atherosclerosis and provides new insights into the interaction between inflammation and cholesterol transport.

## Introduction

Atherosclerosis, a chronic inflammatory disease characterized by fibrofatty lesions formed in the artery walls, is considered a major cause of myocardial infarction (MI) and stroke worldwide (Libby et al., 2019). Foam cell formation is a hallmark of atherosclerosis progression. Most foam cells are cholesterol ester-enriched macrophages originating from monocytes, while others are macrophage-like cells originating from vascular smooth muscle cells (Ouimet et al., 2019). The formation of foam cells is determined by the integrated effect of lipid uptake, cholesterol esterification, and cholesterol efflux (Wang D. et al., 2019).

Therapeutically targeting proinflammatory cytokines is a potential method to tackle atherosclerosis. Cytokines such as IFN-γ, TNF-α, and IL-1β are promoters of macrophage foam cell formation (McLaren et al., 2011), and activated macrophages can exacerbate inflammatory responses by releasing proinflammatory cytokines. In apolipoprotein E-deficient (*ApoE*^–/–^) mice, neutralizing IL-1β significantly reduces the area of aortic atheroma, increase the plaque-free lumen area, and shift blood monocytes to a less inflammatory state (Bhaskar et al., 2011; Vromman et al., 2019). Several clinical studies concerning blocking IL-1β to prevent cardiovascular diseases have been conducted, although none has an indication for large-scale clinical use currently (Buckley and Abbate, 2018). Canakinumab (Ilaris), a fully humanized mAb against IL-1β, significantly lowered the rate of recurrent cardiovascular events without interfering with lipid levels (Ridker et al., 2017), indicating that IL-1β mAb is a promising antiatherosclerotic drug, although the mechanism is not thoroughly understood.

Circular RNAs (circRNAs) are a subclass of non-coding RNAs (ncRNAs) characterized by a stable structure without free 5′ or 3′ ends. circRNAs can function by modulating the activity of microRNAs (miRNAs) as competing endogenous RNAs (ceRNAs), regulating RNA-binding proteins, and could be directly translated into proteins (Han et al., 2018; Xiao et al., 2020). Previous studies have indicated that circRNAs are highly associated with the progression of atherosclerosis. The plasma level of hsa_circ_0001445 is negatively related to coronary atherosclerotic burden in patients (Vilades et al., 2020). The combination of hsa_circ_0001879 and hsa_circ_0004104 is capable of differentiating CAD patients (Wang L. et al., 2019). As an antiatherogenic circRNA, circANRIL modulates ribosomal RNA maturation and influences atherogenic pathways (Holdt et al., 2016). circCHFR and hsa_circ_0029589 were reported to participate in atherogenesis by promoting the proliferation and migration of vascular smooth muscle cells (Yang et al., 2019; Huang et al., 2020). In oxidized low-density lipoprotein (ox-LDL)-treated human aorta endothelial cells (HAECs), circ_0003204 sponges miR-370 and increases TGFβR2 expression, thus inhibiting the proliferation, migration, and tube formation of HAECs (Zhang et al., 2020). To summarize, these results imply that circRNAs play a crucial role in regulating atherosclerosis.

To date, whether circRNAs can be used in anti-atherosclerotic therapies is widely unknown. In the current study, we revealed that a novel circRNA, circDENND1B, could alleviate foam cell formation, and is involved in the antiatherosclerotic effect of IL-1β mAb in mice. circDENND1B could sponge miR-17-5p and increase ATP binding cassette subfamily A member 1 (*Abca1*) expression, thus inhibiting atherosclerosis by promoting cholesterol efflux.

## Materials and Methods

### Animal Experiments

All animal experiments were approved by the ethics committee of Zhongshan Hospital, Fudan University, and the experiments took place in the animal laboratory of Zhongshan Hospital, Fudan University (Shanghai, China). Six-week-old (20.7 ± 2 g) male apolipoprotein E-deficient (*ApoE*^–/–^) mice on a C57BL/6 background were purchased from GemPharmatech (China). The mice were randomly allocated to the experimental group or control group and were fed an HFD with 40% kcal fat, 1.25% cholesterol and 0.5% cholic acid (Research Diets, United States) or chow diet for 12 weeks. For the study involving IL-1β mAb, mice were treated with InVivoMab anti-mouse/rat IL-1β (i.p., twice weekly, 1.0 mg/kg, Bio X Cell, United States) or PBS (i.p., twice weekly, 0.1 ml) from the seventh week of atherogenic diet for 6 weeks. The mice were maintained on a 12:12 h light/dark cycle, with controlled temperature (22 ± 2°C) and humidity (55 ± 5%) conditions, and had free access to water and food. At the end of the 12-week-period, all mice were anesthetized by inhaling isoflurane and euthanized by collecting blood through cardiac puncture.

### Cell Culture

The RAW264.7 cell line was purchased from Shanghai Zhong Qiao Xin Zhou Biotechnology Co., Ltd. (China) and cultured in DMEM (Gibco, United States) supplemented with 10% fetal bovine serum (HyClone, United States) and penicillin/streptomycin solution (HyClone, United States). For foam cell induction, RAW264.7 cells were incubated with 100 mg/ml ox-LDL (Yiyuan Biotech, China) for 48 h.

### Total RNA/circRNA/miRNA Isolation

Total RNA was isolated and purified from cell or tissue samples using TRIzol reagent (Invitrogen, United States) following the manufacturer‘s protocol. RNase R (Lucigen, United States) was used to isolate circRNA from total RNA. The miRcute miRNA Isolation Kit (Tiangen, China) was utilized to isolate miRNA. The concentration and purity of each isolated RNA sample were confirmed with a NanoDrop 2000 (Thermo Fisher Scientific, United States).

### RNA Library Construction, Sequencing and Data Analyses

Approximately 5 μg of total RNA was used in the RNA sequencing assay. Ribosomal RNA was depleted using the Ribo-Zero^TM^ rRNA Removal Kit (Illumina, United States), and the remaining RNA was fragmented into small pieces with divalent cations. We reverse-transcribed the cleaved RNA fragments into cDNA and synthesized U-labeled second-strand DNA with reverse-transcribed cDNA, *E. coli* DNA polymerase I, RNase H and dUTP. An A-base was then added to the blunt ends of each strand, preparing for ligation to the indexed adapters. Single- or dual-index adapters were ligated to the fragments, and AMPureXP beads were used for size selection. After heat-labile UDG enzyme treatment of the U-labeled second-strand DNA, the ligated products were amplified by PCR. The average insert size for the final cDNA library was 300 bp (±50 bp). Finally, we performed paired-end sequencing on an Illumina HiSeq 4000 (LC Bio, China) following the manufacturer’s recommended protocol. To analyze the sequencing data, Cutadapt (Martin, 2011) was used to remove the reads that contained adaptor contamination, low-quality bases or undetermined bases. The sequence quality was then verified with Fast QC. CIRCExplorer (Zhang et al., 2016) and CIRI (Gao et al., 2015) were used to assemble the mapped reads to circRNAs, and the back-splicing reads were identified in the unmapped reads by TopHat-fusion. The differentially expressed circRNAs were selected with log_2_ (fold change) >1 or log_2_ (fold change) <−1 and with statistical significance (*p*-value < 0.05).

### BODIPY-Cholesterol Efflux Assay

To measure cholesterol efflux in RAW264.7 cells, a stock solution of BODIPY-cholesterol (Sigma, United States) was prepared at 5 mM in DMSO. Cells were loaded with 2.5 μM BODIPY-cholesterol in culture medium for 1 h at 37°C and rinsed twice with MEM (HyClone, United States) supplemented with 10 mM HEPES. Then, the cells were incubated with 10 μg/ml apoA-I (Sangon Biotech, China) or 1% (w/v) cholesterol acid (Sangon Biotech, China) for 4 h at 37°C, and the cell supernatant was filtered with a 0.45 μm filter. The BODIPY fluorescence intensity in the supernatants was measured using a fluorescence spectrophotometer. The images of BODIPY-cholesterol were taken under the same exposure time with a fluorescence microscope (Leica, Germany).

### Reverse Transcription and Quantitative Real-Time PCR (qRT-PCR)

The PrimeScript^TM^ RT reagent Kit with gDNA Eraser (TAKARA, China) was used to synthesize cDNA from isolated total RNA or circRNA, and the cDNA was then processed for qRT-PCR using SYBR^®^ Premix Ex Taq^TM^ (TAKARA, China) following the manufacturer’s protocols. Reverse transcription and quantitative detection of miRNA were performed with the miRcute Plus miRNA First-Strand cDNA Kit (Tiangen, China) and miRcute Plus miRNA qPCR Kit (SYBR Green) (Tiangen, China) following the manufacturer’s instructions. Primer sequences are available in Supplementary Table 1.

### Generation of Overexpressing Cell Lines

Lentiviruses overexpressing circDENND1B or containing the control vector were purchased from Hanbio Biotechnology (China). RAW264.7 cells were cultured in 6-well plates, and culture medium containing lentivirus was added when the cells reached 70% confluence. The culture medium was replaced 24 h later, and stable cell lines were selected using puromycin (2 mg/ml, Beyotime, China).

### siRNA and miRNA Mimic Transfection

siRNA targeting circDENND1B, non-specific negative control siRNA, miRNA-17-5p mimics and negative control mimics were purchased from GenePharma (China). Transfection of siRNAs or miRNA mimics into RAW264.7 cells were conducted with Lipofectamine RNAiMAX Transfection Reagent (Thermo Fisher Scientific, United States) according to the manufacturer’s protocols. Six hours after transfection, the medium was replaced. The sequences of the siRNAs and miRNA mimics are provided in Supplementary Table 2.

### Oil Red O Staining

For the Oil Red O (ORO) staining of cells, 24 h after siRNA or miRNA-mimic treatment, RAW264.7 cells were incubated with ox-LDL for 48 h. The cells were washed with PBS and fixed with 4% paraformaldehyde (PFA) for 20 min at room temperature. Then, the cells were incubated with filtered ORO staining solution (Sangon Biotech, China) for 30 min at room temperature and processed for hematoxylin staining for 5 min. For the staining of aorta roots, mouse aorta root tissues were immediately snap-frozen in liquid nitrogen and placed in OCT cryostat embedding compound (Tissue-Tek, United States) after sacrifice. Frozen aorta root sections were then stained with ORO staining solution. The intracellular lipid droplets were observed and assessed under a bright-field microscope (Leica, Germany).

### Western Blot (WB)

Forty-eight hours after ox-LDL and IL-1β mAb treatment, cells were lysed with ice-cold RIPA lysis buffer (Beyotime, China) following the manufacturer’s instructions. The concentration of protein was measured using a BCA Protein Assay Kit (Beyotime, China). Extracted proteins were separated by SDS-PAGE, transferred to polyvinylidene fluoride (PVDF) membranes (Millipore), blocked in 5% BSA in 0.05% Tween 20/TBS for 2 h, and incubated overnight with the following primary antibodies at the indicated dilutions: anti-ABCA1 (1:800; Abcam, #66217, United States), anti-LPL (1:1000; Santa Cruz, #sc0373759, United States) and anti-GAPDH (1:2000; Cell Signaling Technology, #5147, United States). PVDF membranes were exposed to horseradish peroxidase (HRP)-conjugated secondary antibodies (Cell Signaling Technology, United States), and signals were detected with the Luminata^TM^ Forte Western HRP Substrate (Millipore, United States).

### ELISA

Mouse IL-1β, IL-6, and TNF-α in lung and liver homogenates were measured by ELISA. All ELISA tests were performed using DuoSet kits (R&D Systems, United States) following the manufacturer’s instructions, and the results were read on an Epoch^TM^ 2 Microplate Spectrophotometer (BioTek, United States). The quantitation of total protein was measured using a BCA Protein Assay Kit (Beyotime, China).

### Fluorescence *in situ* Hybridization (FISH) Assay

The *in vitro* localization of circDENND1B was measured using a FISH kit (RiboBio, China) following the manufacturer’s instructions, and the FISH probe was synthesized by RiboBio. The localization of circDENND1B was detected under a fluorescence microscope (Leica, Germany).

### Immunohistochemistry (IHC)

IL-1β, IL-6 and TNF-α in aorta roots were measured by IHC. The aorta root sections were immersed in 4% PFA and embedded into paraffin blocks. After dewaxing, the sections were rinsed with PBS, followed by antigen retrieval. After treatment with 3% H2O2 for 25 min, 3% BSA was used to prevent non-specific antibody binding for 30 min, followed by incubation with the following primary antibodies at 4°C overnight: rabbit anti-mouse IL-1β pAb (Abcam, #205924, United States); rabbit anti-mouse IL-6 pAb (Abcam, #208113, United States); and mouse anti-mouse TNF-α mAb (Abcam, #1793, United States). The sections were washed with PBS and then incubated with secondary antibodies for 50 min. Next, DAB buffer was added for development, followed by hematoxylin counterstaining, HCl differentiation, water rinsing and drying in gradient ethanol. The samples were immersed in xylene and mounted with neutral buffered resin. Images were analyzed with ImageJ software.

### RNA Pull-Down Assay

For the RNA pull-down assay, the specific biotinylated probe for circDENND1B was designed and synthesized by Cloudseq (China). Cell lysate was incubated with 3 μg biotinylated probes at room temperature for 1 h. Then, the biotin-coupled RNA complex was pulled down by incubating with streptavidin magnetic beads (Thermo Fisher, United States) for 2 h. Afterward, the magnetic beads were washed with lysis buffer five times, and the samples were resuspended in 50 μl elution buffer, followed by RNA extraction and qRT-PCR assay of the target miRNA.

### Statistical Analysis

Statistical analyses were performed with Prism7 (GraphPad, United States). Unpaired two-tailed *t*-test was used to compare data between two groups. For experiments involving more than two groups, data were analyzed with one-way ANOVA followed by Dunnett’s *post hoc* test. Data are presented as the mean ± SEM unless otherwise mentioned. *P*-values < 0.05 were considered statistically significant.

## Results

### A High-Fat Diet Induced an Inflammatory State in *ApoE*^–/–^ Mice

Six-week-old male *ApoE*^–/–^ mice were randomly allocated to the chow diet group or high-fat diet (HFD) group. After an HFD for 12 weeks, atherosclerotic lesions could be detected in the aorta, and the lesion area was significantly larger in mice fed an HFD than in mice fed a normal diet (Supplementary Figures 1A–C).

To confirm that HFD-fed mice were in a systemic inflammatory state (Bäck et al., 2019), ELISA was conducted to measure the levels of IL-1β, IL-6, and TNF-α in the lungs and livers, which showed that the HFD-fed mice were in a systemic inflammatory state (Supplementary Figures 2A–F). The immunohistochemistry (IHC) positive staining areas of IL-1β, IL-6, and TNF-α in the aortic roots were also larger in atherosclerotic mice (Supplementary Figure 2G). In addition, the expression of IL-1β and IL-6 mRNA clearly increased in atherosclerotic aortas, while the expression of TNF-α in aortas increased but did not reach statistical significance (Supplementary Figure 2H). IL-1β and TNF-α were significantly highly expressed in the lungs and livers of atherosclerotic mice (Supplementary Figures 2I,J). The above results indicate that HFD-fed mice were in a highly inflammatory state, which stresses the necessity of anti-inflammation therapy toward atherosclerosis.

### Differentially Expressed mRNA/circRNA in Atherosclerotic *ApoE*^–/–^ Mice Were Identified With mRNA/circRNA Sequencing

To investigate the roles of circRNAs in the initialization and development of atherosclerotic plaques, we extracted RNA from the aortas of chow diet-fed or HFD-fed mice and conducted second-generation sequencing to identify the differentially expressed mRNAs and circRNAs.

**FIGURE 1:**
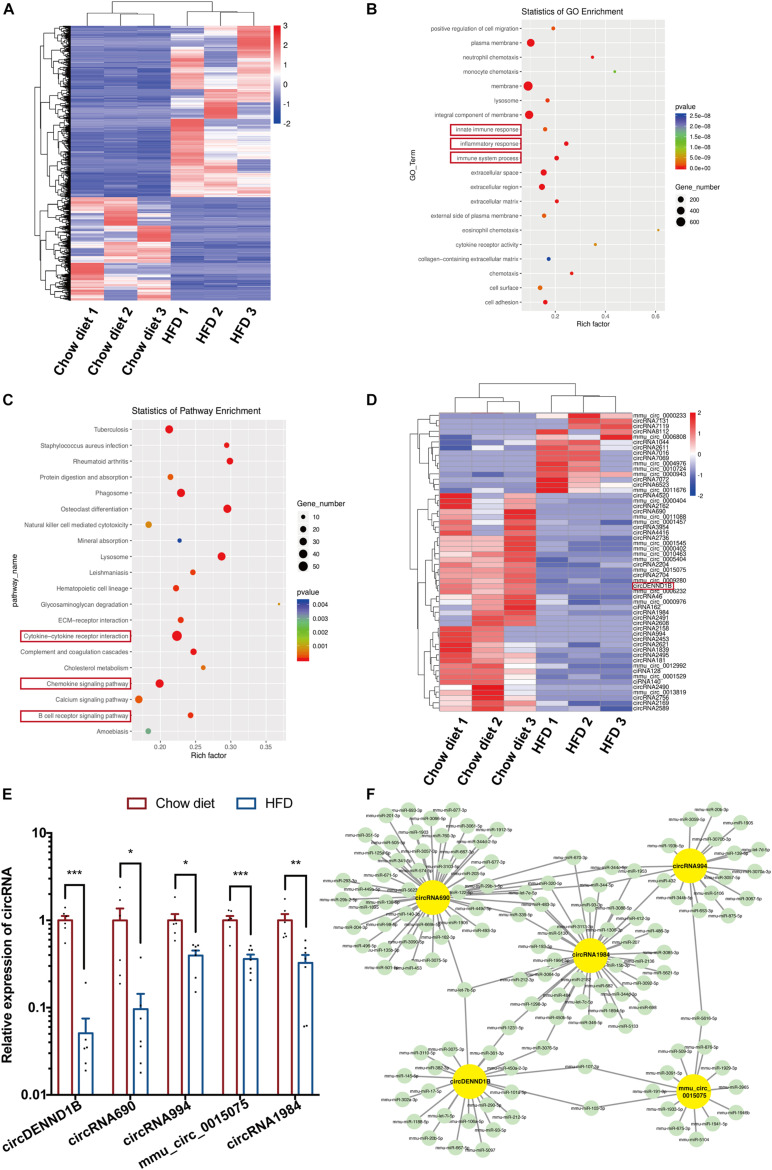
Differentially expressed circular RNA/mRNA in mouse aortas. **(A)** Heatmap of DEGs in the aortas of HFD-fed compared to chow diet-fed mice. **(B)** The top 20 GO terms of detected DEGs. The enriched GO terms are ordered alphabetically. **(C)** Scatterplots of the top 20 differentially regulated pathways identified in KEGG. The KEGG terms are ordered alphabetically. **(D)** Heatmap of the 56 identified DECs in mouse aortas. **(E)** qRT-PCR results confirming that five DECs are downregulated in atherosclerotic aortas, Data were normalized to GAPDH (*n* = 6 per group). **(F)** The ceRNA module network of five identified DECs. The yellow nodes represent circRNAs, and the green nodes represent predicted miRNAs. *p < 0.05, **p < 0.01, ***p < 0.001.

From the mRNA sequencing results, 1732 upregulated genes and 1047 downregulated genes were identified (*p* < 0.05) (Figure 1A). Gene ontology (GO) analyses of the differentially expressed genes (DEGs) revealed that innate immune response, inflammatory response, and immune system process were significantly affected (Figure 1B). Kyoto Encyclopedia of Genes and Genomes (KEGG) analyses showed that the DEGs were significantly enriched in pathways related to inflammation, including the chemokine signaling pathway, cytokine-cytokine receptor interaction, and B cell receptor signaling pathway (Figure 1C). These results confirmed that modulating inflammation is a promising strategy for the treatment of atherosclerosis.

Furthermore, 56 differentially expressed circRNAs (DECs) were identified out of 10024 circRNAs. The numbers of increased and decreased circRNAs in HFD-fed mice were 15 and 41, respectively (Figure 1D). Among the DECs with a *P* value lower than 0.01, we confirmed that the expression of circDENND1B, circRNA690, circRNA994, mmu-circ-0015075 and circRNA1984 decreased in the aortas of HFD-fed mice (Figure 1E). The selected DECs target over 100 miRNAs, as predicted by RegRNA (Chang et al., 2013) (Figure 1F).

### circDENND1B Participates in the Antiatherosclerotic Effect of IL-1β mAb *in vitro*, and Its Expression Was Affected by IL-1β mAb *in vivo*

**FIGURE 2:**
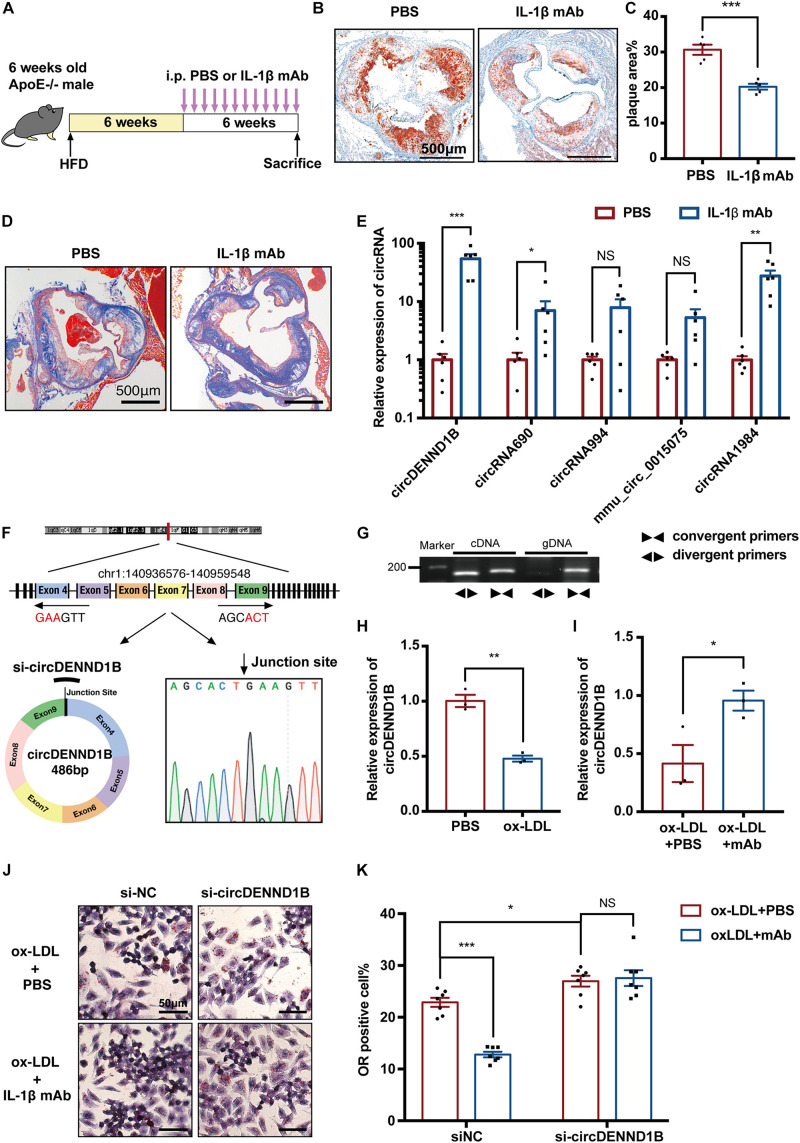
circDENND1B participates in the antiatherosclerotic effect of IL-1β mAb *in vitro*, and its expression was affected by IL-1β mAb *in vivo.* **(A)** Schematic depiction of the study in which ApoE^– /–^ mice were fed a HFD for 12 weeks and treated with PBS or IL-1β mAb. **(B)** ORO staining showing that IL-1β mAb injection resulted in a smaller plaque area in the aortic roots. **(C)** Quantification of the percentage of plaque area compared to the total area of aortic roots (*n* = 5 per group). **(D)** Representative picture of Masson’s trichrome staining of aorta roots. **(E)** The expression of five circRNAs in aortas was detected with qRT-PCR. Data were normalized to GAPDH (*n* = 6 per group). **(F,G)** Schematic depiction of circDENND1B’s structure, showing it is comprised of the fourth to ninth exon of *Dennd1b*. Agarose gel electrophoresis showing that circDENND1B is a circular RNA. The circRNA was amplified by divergent primers (◀▶) from cDNA but not from gDNA. *Dennd1b*, the host gene of circDENND1B, was amplified by convergent primers (▶◀) from both cDNA and gDNA. Sanger sequencing validated its splicing junction. **(H)** qRT-PCR shows that circDENND1B expression decreased in RAW264.7 cells treated with ox-LDL. **(I)** qRT-PCR shows that circDENND1B expression increased after IL-1β mAb treatment. **(J)** Representative image of ORO staining, depicting that fewer foam cells are formed in RAW264.7 cells treated with ox-LDL and IL-1β mAb. **(K)** The percentage of foam cells was lower after IL-1β mAb treatment. Approximately 1000 cells were counted per treatment over seven separate experiments. gDNA, genomic DNA; NS, no significant difference, *p < 0.05, **p < 0.01, ***p < 0.001.

As atherosclerotic mice were in an inflammatory state, we wondered whether blocking certain cytokines could affect atherosclerosis via modulation of circRNAs. Thus, we intraperitoneally injected IL-1β mAb, which was proven effective in preventing MI in the CANTOS study (Ridker et al., 2017), into HFD-fed *ApoE*^–/–^ male mice biweekly (Figure 2A). IL-1β mAb treatment relieved atherosclerotic plaques and attenuated local inflammation in aortic roots (Figures 2B,C and Supplementary Figures 3A,B). Besides, the level of collagen around atherosclerotic plaques was detected using Masson’ trichrome staining, indicating that the plaque in IL-1β mAb-treated mice is more stable (Figure 2D).

To determine the participation of circRNAs in the antiatherosclerotic process, we conducted qRT-PCR assay on circRNAs. Among the DECs identified between the aortas of HFD-fed and chow diet-fed mice, the expression of circDENND1B increased markedly after IL-1β mAb treatment (Figure 2E), suggesting the possibility of association between circDENND1B and the antiatherosclerotic effect of IL-1β mAb *in vivo*.

Furthermore, we established an *in vitro* foam cell model by incubating RAW264.7 cells with 100 mg/ml ox-LDL for 48 h. Foam cell formation was demonstrated by ORO staining (Supplementary Figure 4A). We confirmed the structure of circDENND1B using PCR, agarose gel electrophoresis, and Sanger’s sequencing (Figures 2F,G). The expression of circDENND1B was downregulated in ox-LDL-treated cells (Figure 2H). In cells incubated with IL-1β mAb, while the expression of circDENND1B was upregulated (Figure 2I), an alleviating trend in lipid accumulation was observed, indicating that IL-1β mAb could interfere with lipid accumulation or efflux (Figures 2J,K). Moreover, although IL-1β mAb treatment significantly alleviated foam cell formation, this effect was inhibited after knocking down circDENND1B, indicating that circDENND1B is necessary in the anti-foam-cell-formation process of IL-1β mAb. These results suggest that circDENND1B protects atherogenesis, and participates in the antiatherosclerotic effect of IL-1β mAb *in vitro*.

### circDENND1B Inhibits Foam Cell Formation by Promoting *Abca1* Expression

**FIGURE 3:**
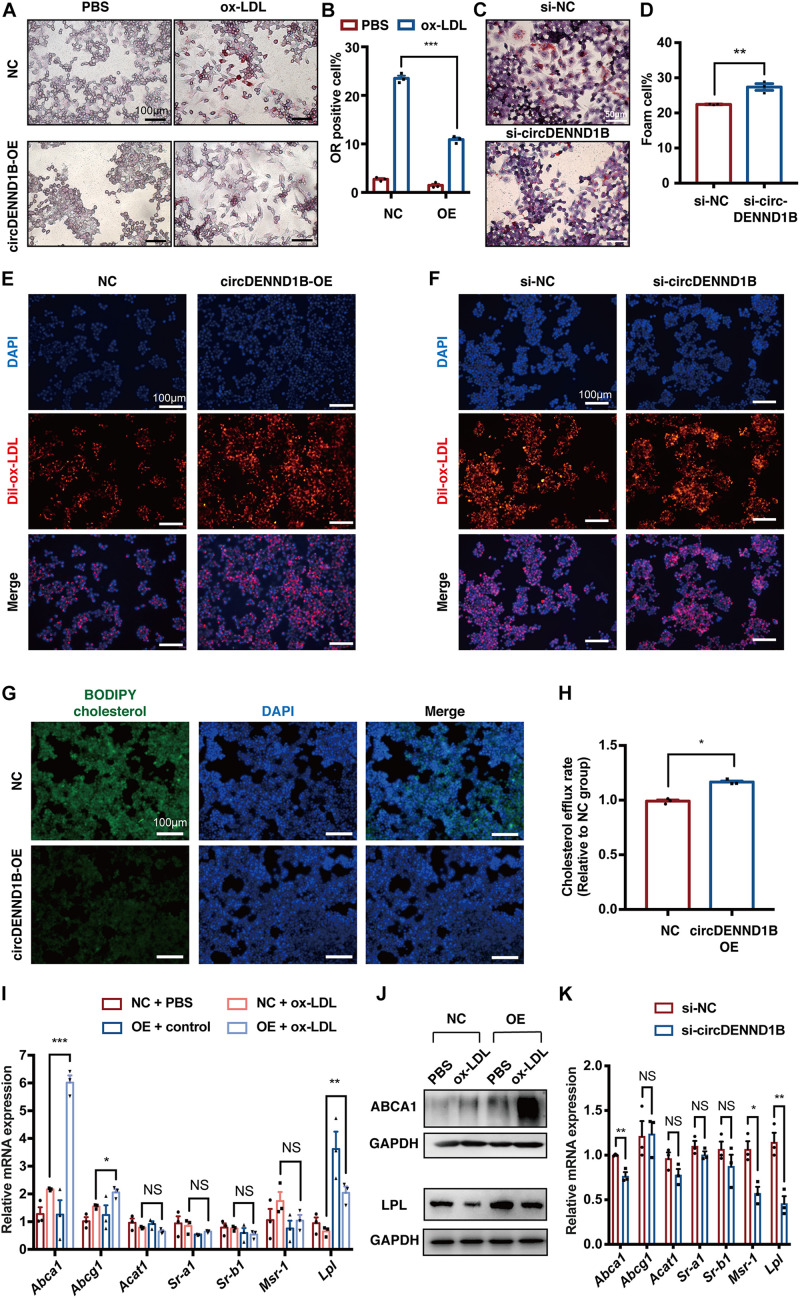
circDENND1B regulates foam cell formation by promoting cholesterol efflux. **(A)** ORO staining showing that fewer foam cells are formed in RAW264.7 cells overexpressing circDENND1B. **(B)** The percentage of foam cells was lower after overexpressing circDENND1B. Approximately 1000 cells were counted per treatment over three separate experiments. **(C)** ORO staining showing that fewer foam cells are formed after knocking down circDENND1B. **(D)** The percentage of foam cells was higher after knocking down circDENND1B. Approximately 1000 cells were counted per treatment over three separate experiments. **(E,F)** Representative fluorescent images of cells treated with dil-ox-LDL for 48 h. Dil-ox-LDL positive cells were stained in red. **(G)** Representative images of the BODIPY assay showing overexpressing circDENND1B lowered cellular cholesterol. **(H)** Relative cholesterol efflux rate in cells overexpressing circDENND1B, measured by BODIPY assay. **(I)** qRT-PCR results of genes related to foam cell formation. Data were normalized to GAPDH (*n* = 3 per group). **(J)** WB results of proteins related to foam cell formation after ox-LDL treatment. **(K)** qRT-PCR results of genes related to foam cell formation in RAW264.7 cells. Data were normalized to GAPDH (*n* = 3 per group). NC: negative control. OE, overexpress; NS, no significant difference, *p < 0.05, **p < 0.01, ***p < 0.001.

To explore the possible roles of circDENND1B in the formation of macrophage-derived foam cells, we used siRNA to knock down circDENND1B, and established a circDENND1B-overexpressing RAW264.7 cell line using lentivirus (Supplementary Figures 5A,B). Cells overexpressing circDENND1B exhibit a significantly lower percentage of ORO-positive cells, while circDENND1B-knock down cells show an opposite trend (Figures 3A–D), indicating that circDENND1B regulates foam cell formation.

Foam cell formation is mainly determined by the combined effect of lipid uptake and cholesterol efflux (Wang D. et al., 2019). As illustrated in Figures 3E,F, the uptake of ox-LDL remains unchanged in cells overexpressing circDENND1B or transfected with si-circDENND1B, as compared with control group. However, the cholesterol efflux rate significantly increased in OE group (Figures 3G,H), suggesting that circDENND1B possibly regulates foam cell formation by promoting cholesterol efflux. Therefore, we conducted qRT-PCR on genes related to foam cell formation, including *Abca1*, *Abcg1*, *Acat1*, *Sr-a1*, *Sr-b1*, *Msr-1, and Lpl* (McLaren et al., 2011). In cells treated with ox-LDL, *Abca1* expression increased significantly after overexpressing circDENND1B, which is the most significant among these genes (Figure 3I). Moreover, the increase of ABCA1 protein expression after circDENND1B overexpression was further identified using WB (Figure 3J). On the contrary, the expression of *Abca1*, as well as *Msr-1* and *Lpl*, decreased in cells transfected with si-circ-DENND1B (Figure 3K). These results suggested that circDENND1B modulates foam cell formation via promoting cholesterol efflux, and *Abca1* is a potential target of circDENND1B.

### circDENND1B Participates in the Antiatherosclerotic Effect of IL-1β mAb by Promoting Cholesterol Efflux

**FIGURE 4:**
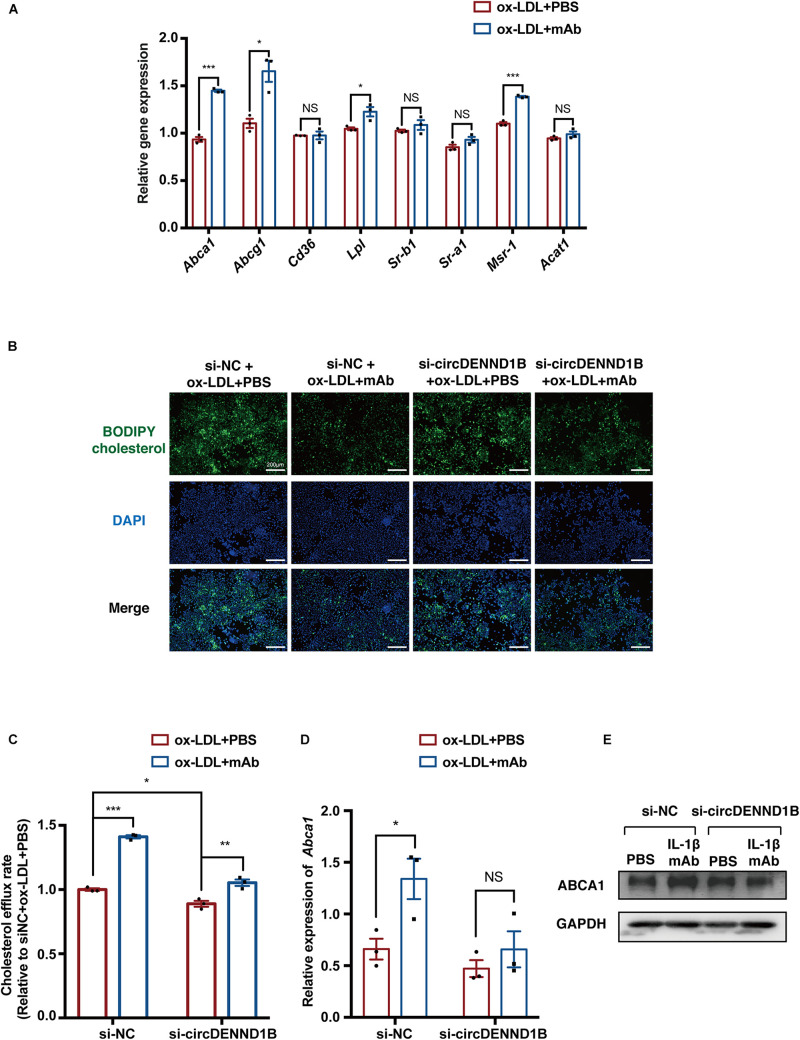
circDENND1B is crucial in the pro-cholesterol efflux effect of IL-1β mAb *in vitro.* **(A)** qRT-PCR results of genes related to foam cell formation in IL-1β mAb-treated foam cells. **(B)** Representative images of BODIPY assay showing circDENND1B mediates the pro-cholesterol efflux effect of IL-1β mAb. **(C)** Relative cholesterol efflux rate in RAW264.7 cells, measured by BODIPY assay (*n* = 3 per group). **(D)** qRT-PCR results of *Abca1* expression in si-circDENND1B-treated cells, data were normalized to GAPDH data (*n* = 3 per group) **(E)** WB results of ABCA1 in cells transfected with si-circDENND1B and treated with ox-LDL plus PBS or IL-1β mAb.

As circDENND1B is correlated with the antiatherosclerotic effect of IL-1β mAb, we then conducted experiments to identify whether this effect is also related to cholesterol efflux. From qRT-PCR results on genes related to foam cell formation, we found that *Abca1* was most significantly differentially regulated in cells treated with IL-1β mAb and ox-LDL (Figure 4A), suggesting that IL-1β mAb is capable of mediating *Abca1*.

Moreover, in si-circDENND1B-treated cells, the increase in cholesterol efflux induced by IL-1β mAb was less significant than in the si-NC group (Figures 4B,C) (Sankaranarayanan et al., 2011). qRT-PCR and Western blotting also showed that the increase of *Abca1* mRNA expression and ABCA1 protein expression induced by IL-1β mAb is compromised after knocking down circDENND1B (Figures 4D,E). These data suggested that circDENND1B modulates the antiatherosclerotic effect of IL-1β mAb by promoting cholesterol efflux.

### circDENND1B/miR-17-5p/*Abca1* Regulates the Inhibition of Foam Cell Formation Induced by IL-1β mAb

**FIGURE 5:**
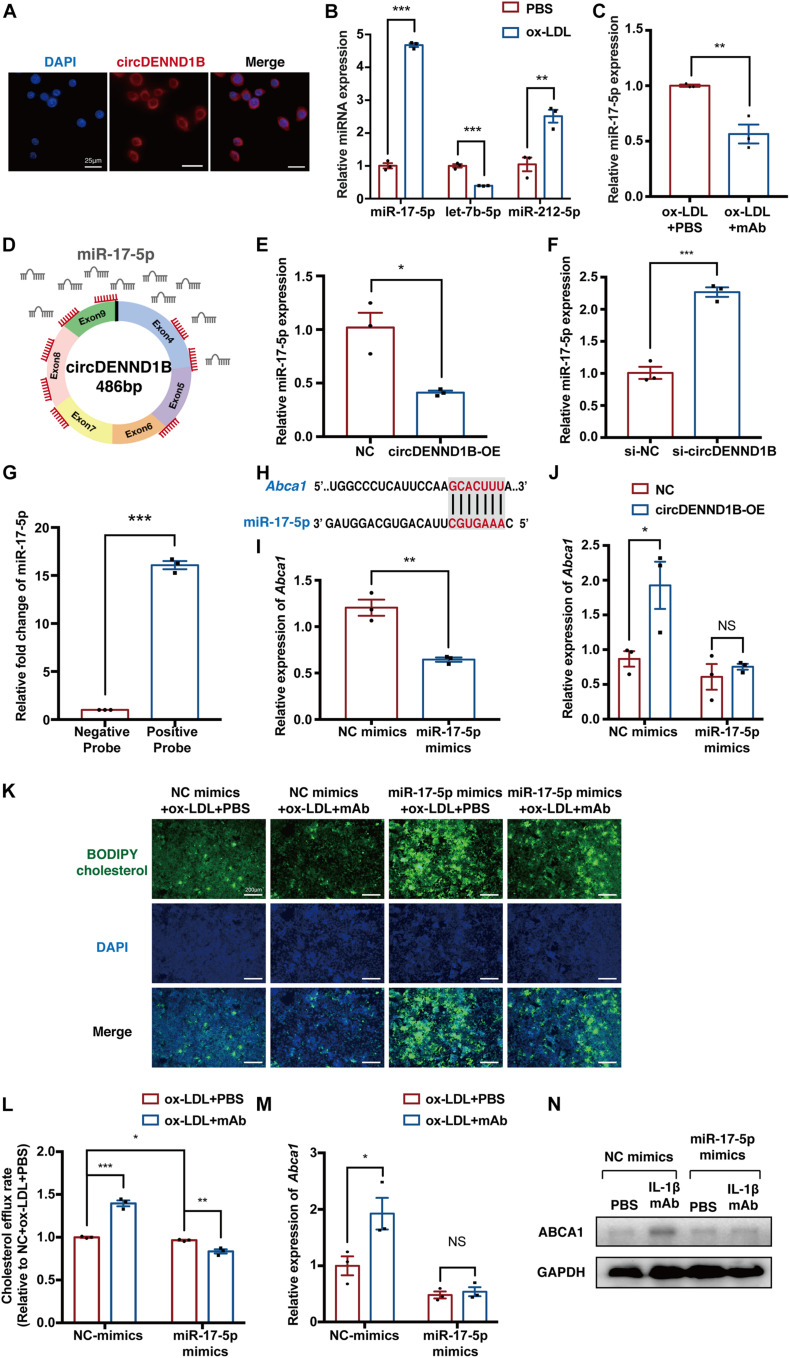
circDENND1B/miR-17-5p/*Abca1* inhibits foam cell formation induced by IL-1β mAb. **(A)** Fluorescence *in situ* hybridization (FISH) assay showing that circDENND1B is localized in the cytoplasm. **(B)** qRT-PCR results of let-7b-5p, miR-17-5p and miR-212-5p in ox-LDL-treated cells. **(C)** qRT-PCR results of miR-17-5p in cells treated with ox-LDL and PBS or IL-1β mAb. **(D)** Schematic of the miR-17-5p sponge mechanism of circDENND1B, showing eight binding sites (red) of miR-17-5p (gray) in circDENND1B. **(E)** qRT-PCR shows miR-17-5p expression decreased after overexpressing circDENND1B. **(F)** qRT-PCR shows miR-17-5p expression increased after knocking down circDENND1B. **(G)** RNA pull-down assay confirmed the binding between circDENND1B and miR-17-5p. **(H)** miR-17-5p was predicted to bind the 3′UTR of *Abca1*. **(I)** qRT-PCR results of *Abca1* in foam cells treated with NC mimics or miR-17-5p mimics. **(J)** qRT-PCR results of *Abca1* in cells transfected with miRNA mimics and overexpressing circDENND1B. **(K)** Representative images of the BODIPY assay showing the impact of miR-17-5p mimics and IL-1β mAb on cholesterol efflux. **(L)** Cholesterol efflux rate in miR-17-5p-mimics-treated cells, measured by BODIPY assay. **(M)** qRT-PCR results of *Abca1* in cells transfected with miRNA mimics (*n* = 3 per group). **(N)** WB results of ABCA1 in cells transfected with miR-17-5p and treated with ox-LDL plus PBS or IL-1β mAb. The qRT-PCR data of mRNA were normalized to GAPDH data (*n* = 3 per group), and miRNA expression levels were normalized to U6 levels (*n* = 3 per group). NC, negative control; OE, overexpression; NC mimics, negative control miRNA mimics. *p < 0.05, **p < 0.01, ***p < 0.001.

MicroRNA sponging is one of the most predominant functions of circRNAs. As circDENND1B is predominantly localized in the cytoplasm of RAW264.7 cells (Figure 5A), which is similar to the location of most miRNAs (Li et al., 2013), we hypothesized that circDENND1B promotes *Abca1* expression through a miRNA sponging mechanism.

To confirm this, we used RegRNA to predict the target miRNAs of circDENND1B. Among the 23 miRNAs predicted to bind with circDENND1B, three miRNAs, let-7b-5p (Wei et al., 2018), miRNA-17-5p (miR-17-5p) (Huang et al., 2018; An et al., 2019; Tan et al., 2019) and miRNA-212-5p (miR-212-5p) (Miao et al., 2018), have been reported to affect foam cell formation. The expression of miR-17-5p and miR-212-5p increased in cells treated with ox-LDL, and miR-17-5p decreased after IL-1β mAb treatment (Figures 5B,C). As circDENND1B harbors eight binding sites for miR-17-5p, and miR-17-5p has been reported to modulate the progress of atherosclerosis via *Abca1* (He et al., 2015; Tan et al., 2019; Liu L. et al., 2020) (Figure 5D), we investigated the relationship between circDENND1B and miR-17-5p. Upregulating circDENND1B resulted in a decrease in miR-17-5p expression, while knocking-down had a converse effect (Figures 5E,F). A biotinylated RNA pull-down assay further confirmed that circDENND1B is a miRNA-17-5p sponge (Figure 5G).

In RAW264.7 cells overexpressing miR-17-5p, qRT-PCR results showed a decrease in *Abca1* expression (Supplementary Figure 5C and Figures 5H,I). The increase in *Abca1* induced by overexpressing circDENND1B was compromised in the presence of miR-17-5p mimics (Figure 5J). As Tan et al. (2019) have confirmed the binding property between miR-17-5p and *Abca1*, these findings suggested that circDENND1B sponges miR-17-5p to increase *Abca1* in foam cells.

Next, we identified the role of miR-17-5p in the antiatherosclerotic effect. Overexpressing miR-17-5p with miRNA mimics in foam cells attenuated the IL-1β mAb-induced changes in cholesterol efflux, foam cell formation and *Abca1* expression, suggesting that overexpressing miR-17-5p has a similar influence on foam cell formation as silencing circDENND1B (Figures 5K–N and Supplementary Figures 6A,B).

**FIGURE 6:**
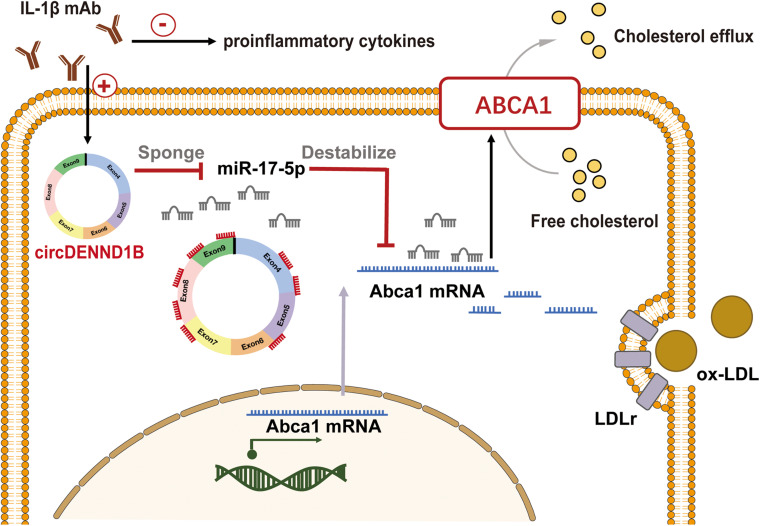
Proposed model of circDENND1B/miR-17-5p/*Abca1* inhibits foam cell formation induced by IL-1β mAb. Treating macrophages with IL-1β mAb may increase the expression of circDENND1B, thus inhibit the miR-17-5p-*Abca1* pathway. The increased ABCA1 protein would promote cholesterol efflux, and regulate the formation of foam cells.

In conclusion, these results indicate that both circDENND1B and miR-17-5p modulate the impact of IL-1β mAb on *Abca1*, thus promoting cholesterol efflux (Figure 6).

## Discussion

Modulating inflammation is crucial in treating atherosclerosis, and blocking proinflammatory cytokines is a promising strategy to prevent atherogenesis. Inflammatory responses influence lipid metabolism in atherosclerosis and vice versa. IL-1β, a soluble cytokine that plays a pivotal role in innate immune responses, was reported to mediate lipid transport by inhibiting ABCA1 (Tumurkhuu et al., 2018; Liu H. et al., 2020), but the mechanism has not been thoroughly elucidated. In this work, we conducted circRNA-sequencing in atherosclerotic mouse aortas for the first time and identified a novel circRNA, circDENND1B, through which IL-1β mAb inhibits atherosclerosis and promotes cholesterol efflux.

circRNAs are a novel subclass of ncRNAs that play important roles in multiple physiological processes, but the roles of circRNAs in atherosclerosis are not well understood. In the present study, we identified 56 DECs in the aortas of HFD-fed and chow-diet-fed mice. Among them, an exonic circRNA, circDENND1B, was shown to modulate *Abca1* expression by sponging miR-17-5p, thus inhibiting foam cell formation. A significant negative correlation between circDENND1B and miR-17-5p was observed by qRT-PCR, suggesting that detectable cellular miR-17-5p decreased after being sponged. Moreover, we confirmed that miR-17-5p is physically bound to circDENND1B using RNA pull-down assay. These results are consistent with previous studies which showed that miR-17-5p accelerates atherogenesis by modulating lipid accumulation (Tan et al., 2019) and endothelial cell ferroptosis (Xiao et al., 2019).

ABCA1 is an essential receptor for the initial step of cholesterol efflux from foam cells in atherosclerotic plaques. Approximately 50% of the net cholesterol efflux from cholesterol-laden mouse macrophages is attributed to ABCA1, while another member of the ATP binding cassette superfamily, ATP binding cassette subfamily G member 1 (ABCG1), accounts for 20% of cholesterol efflux (Adorni et al., 2007). Moreover, various proinflammatory cytokines, including IL-1β, TNF-α, and IFN-γ, can regulate *Abca1* expression and promote foam cell formation (McLaren et al., 2011). Consistent with prior reports showing that IL-1β could suppress *Abca1* expression in macrophages (Tumurkhuu et al., 2018), we confirmed that blocking IL-1β elevated the expression of *Abca1* and promoted cholesterol efflux in foam cells and showed that the pro-cholesterol-efflux function of IL-1β mAb is partially mediated through circDENND1B.

Nevertheless, other possible mechanisms of the antiatherogenic effect of IL-1β mAb should be considered. According to former reports, IL-1β competes for access to ABCA1 with cholesterol (Tumurkhuu et al., 2018) and downregulates ABCA1 through the ROS-NF-κB pathway (Chen et al., 2007). Blocking IL-1β with mAbs disrupts the IL-1β positive feedback loop, thus promoting cholesterol efflux and preventing the production of IL-1β itself. Moreover, IL-1β contributes greatly to smooth muscle cell (SMC) proliferation, endothelial cell activation, and the modulation of various immune cells (Tall and Yvan-Charvet, 2015; Williams et al., 2019). Whether circDENND1B plays a role in these processes still needs further investigation.

A limitation of this study is that the expression and function of circDENND1B were not identified in other species except for mouse, especially in humans. According to previous studies, a proportion of circRNAs are conserved among mammals in organs including the heart, brain, and liver (Rybak-Wolf et al., 2015; Werfel et al., 2016; Xia et al., 2017). In a recent study concerning atherosclerosis (Zeng et al., 2021), circMAP3K5 was found to prevent hyperplasia of vascular smooth muscle cells conservatively in both human and mouse. Although The mouse circRNA circDENND1B was not detected in human macrophages, it was found to be conserved with the human circRNA hsa_circ_0111650 in the brain (Rybak-Wolf et al., 2015), but the effect of the latter circRNA on human foam cell formation is still unknown. Therefore, to prove the therapeutic potential of circDENND1B, further research is necessary to identify whether it could exert its antiatherosclerotic effect in human.

In conclusion, our results suggest that circDENND1B is a ceRNA through which IL-1β mAb inhibits atherosclerosis. This newly identified circRNA could be a promising therapeutic target of atherosclerosis.

## Data Availability Statement

The datasets presented in this study can be found in online repositories. The names of the repository/repositories and accession number(s) can be found below: NCBI GEO (accession: GSE159379).

## Ethics Statement

The animal study was reviewed and approved by The Ethics Committee of Zhongshan Hospital.

## Author Contributions

FX and LS came up with the research idea and designed the experiments. FX, HC, RW, and TZ conducted the experiments and analyzed the data. LS, JQ, and JG supervised the project. All authors read and approved the final manuscript.

## Conflict of Interest

The authors declare that the research was conducted in the absence of any commercial or financial relationships that could be construed as a potential conflict of interest.
