# Supplementary material for: circDENND1B Participates in the Antiatherosclerotic Effect of IL-1β Monoclonal Antibody in Mouse by Promoting Cholesterol Efflux via miR-17-5p/Abca1 Axis
Source: Front Cell Dev Biol. 2021 Apr 29;9:652032. doi: 10.3389/fcell.2021.652032 (PMC8116881; doi:10.3389/fcell.2021.652032)
Supplement: Supplementary file 1 [file Data_Sheet_1.docx]

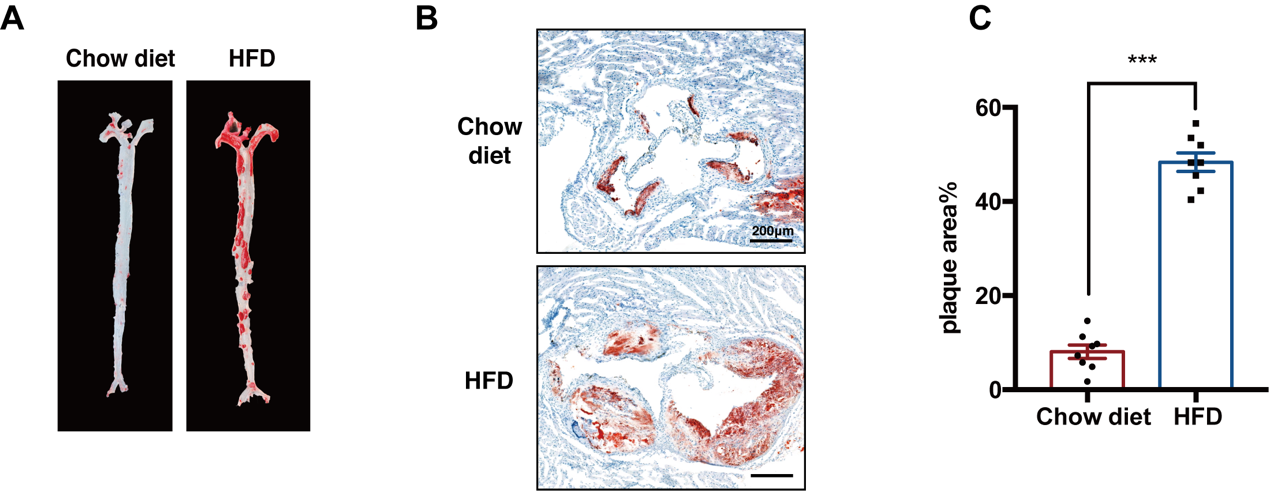
 **Figure S1. Atherosclerotic plaque is formed in HFD-fed-mice** (A) ORO staining showing that HFD resulted in a larger plaque area in the aortas of ApoE-/- mice. (B) ORO staining showing that HFD resulted in a larger lesion area in aortic roots of ApoE-/- mice. (C) Quantification of the percentage of lesion-positive area compared to total area of aortic roots (n=8 per group).


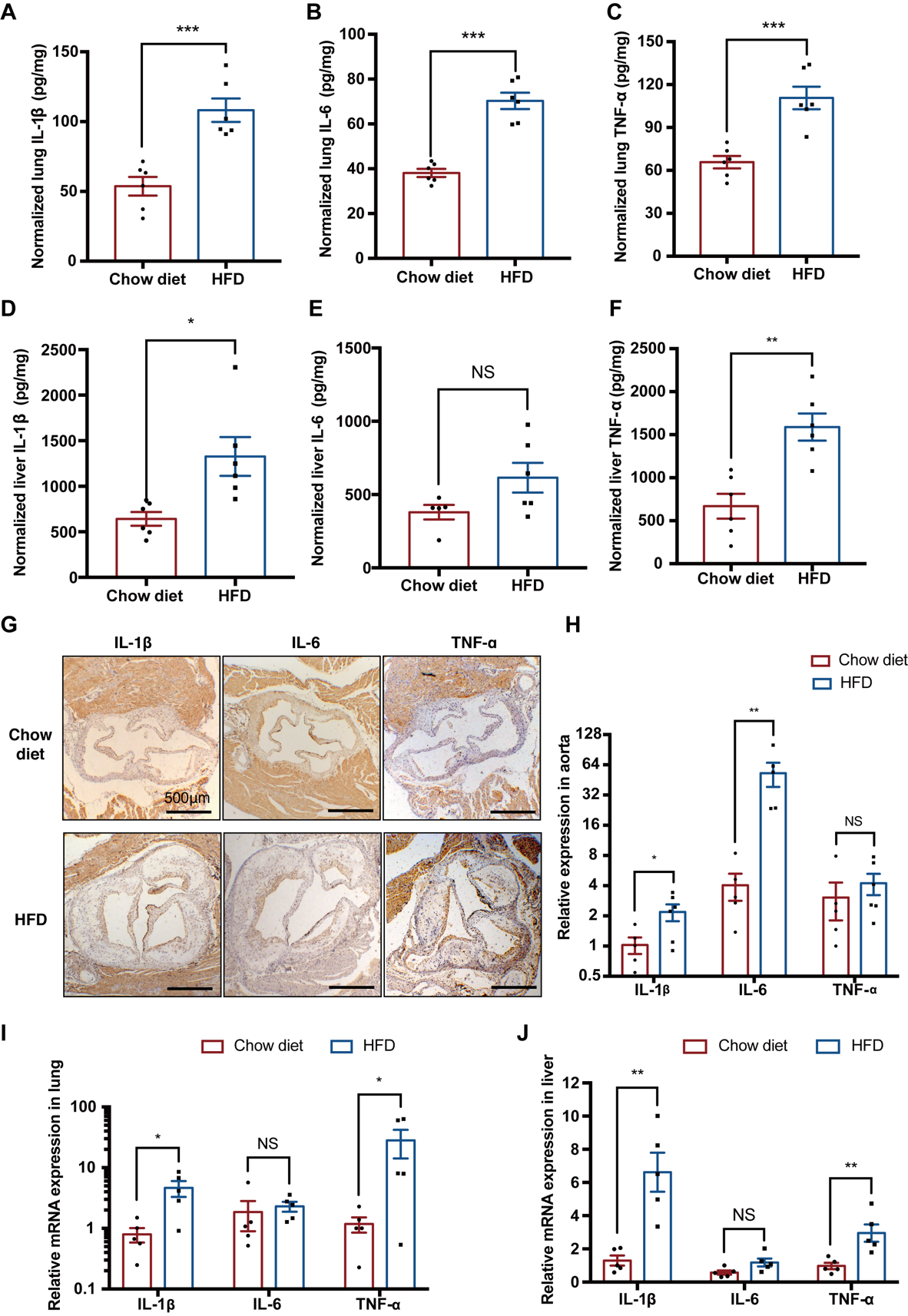


**Figure S2. HFD induced an inflammatory state in ApoE-/- mice** (A-C) ELISA results showing that proinflammatory cytokines are highly expressed in the lungs of HFD-fed mice (n=5-6 per group). (D-F) ELISA results showing that proinflammatory cytokines are highly expressed in the livers of HFD-fed mice (n=5-6 per group). (G) Representative images of IHC staining of aortic roots, suggesting that IL-1β, IL-6 and TNF-α are locally expressed in atherosclerotic mice. (H-J) Relative expression of IL-1β, IL-6 and TNF-α mRNA in the aortas and lungs was measured by qRT-PCR. The gene expression levels were normalized to GAPDH levels (n=5-6 per group). NS: no significant difference, *p<0.05, **p<0.01, ***p<0.001.


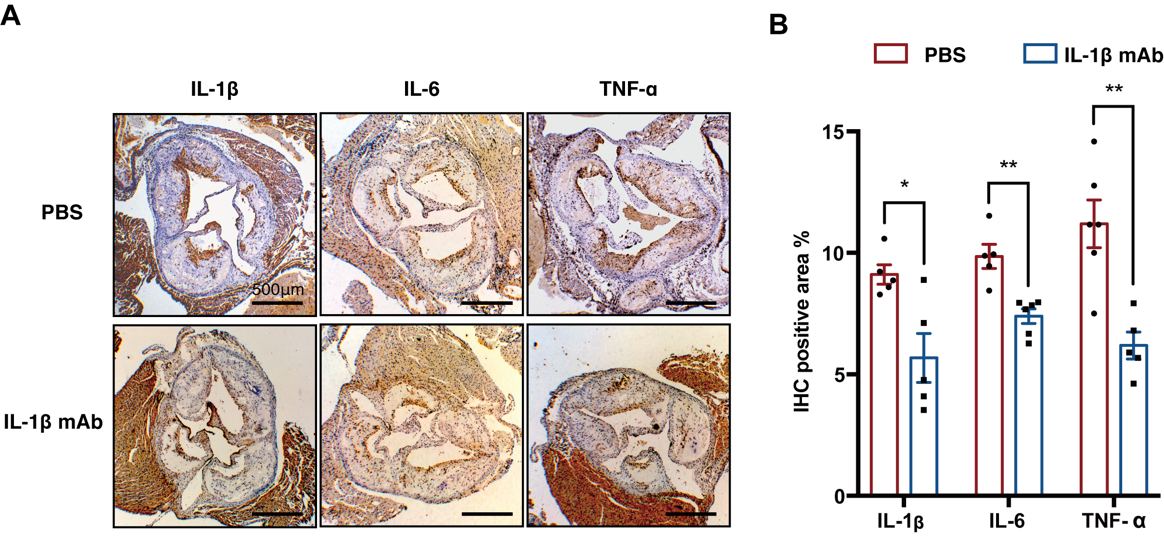


**Figure S3. Local inflammation in aortic roots decreased after IL-1β mAb treatment** (A) Representative IHC images of IL-1β, IL-6 and TNF-α showing that IL-1β mAb treatment lowered local inflammation in aorta roots. (B) Quantification of the percentage of IL-1β-, IL-6- or TNF-α-positive areas in aortic roots (n=5 per group).


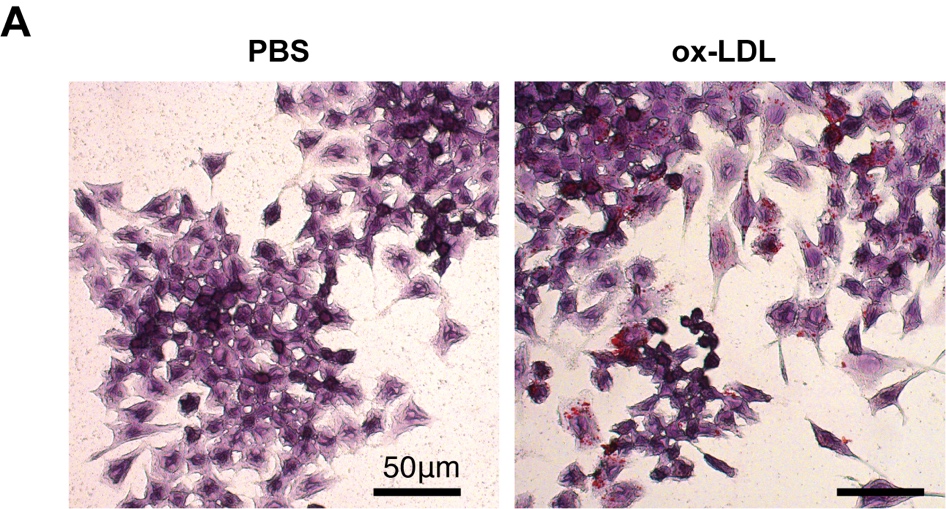


**Figure S4. Foam cell formation *in vitro*** (A) Representative ORO-staining images of RAW264.7 cells treated with ox-LDL, showing that foam cells are formed.


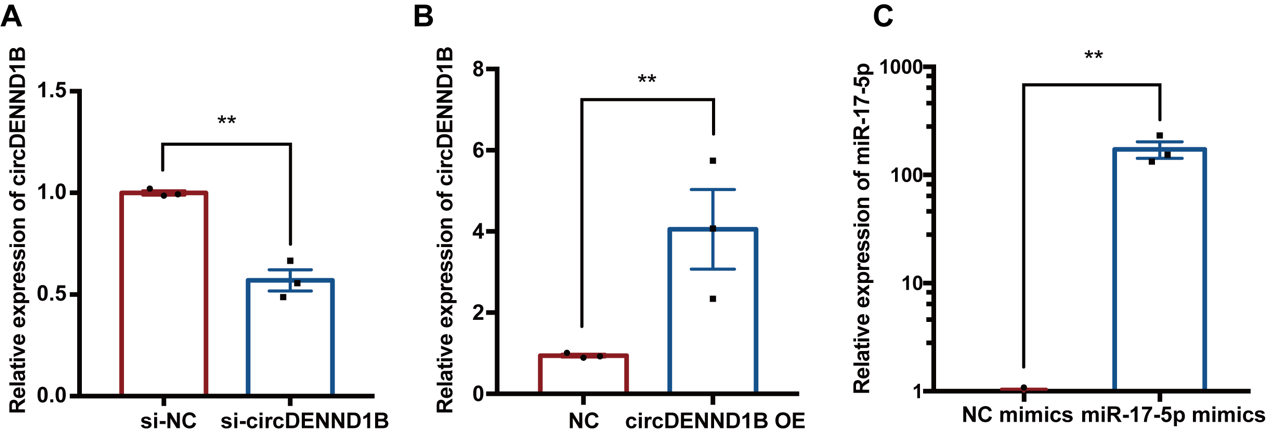


**Figure S5. The efficiency of siRNA and overexpressing-lentivirus targeting circDENND1B, and miR-17-5p mimics** (A) qRT-PCR shows that circDENND1B expression decreased significantly after the transfection of si-circDENND1B. (B) qRT-PCR shows that circDENND1B expression increased significantly after treating cells with circDENND1B-OE lentivirus. (C) qRT-PCR shows that miR-17-5p expression increased significantly after the transfection of miR-17-5p mimics.


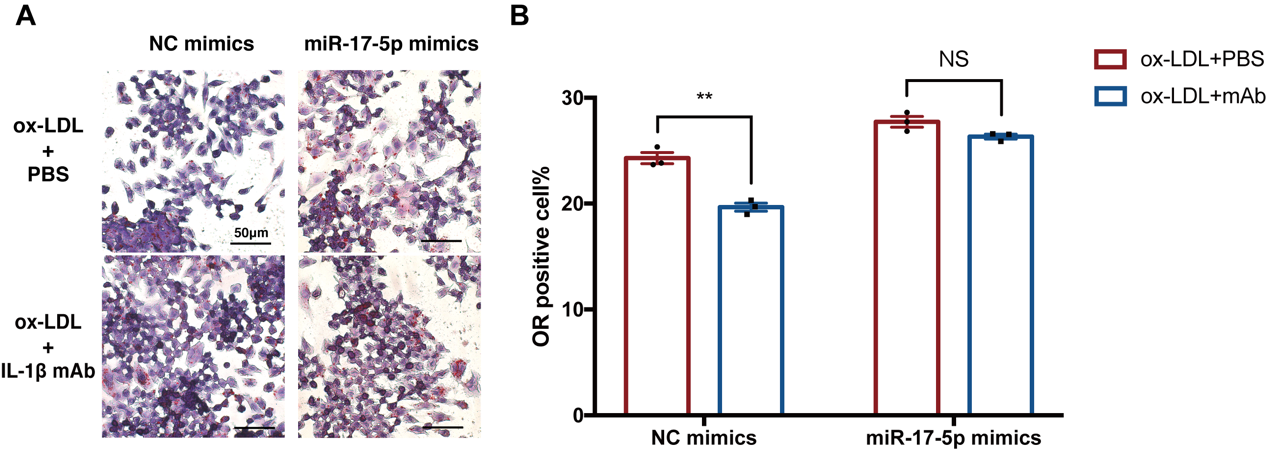


**Figure S6. miR-17-5p modulates the antiatherosclerotic effect of IL-1β mAb.** (A) ORO staining showing that fewer foam cells are formed after overexpressing miR-17-5p, and the inhibition of foam cell formation induced by IL-1β mAb is compromised. (B) Quantification of the percentage of foam cells. Approximately 1000 cells were counted per treatment over 3 separate experiments.


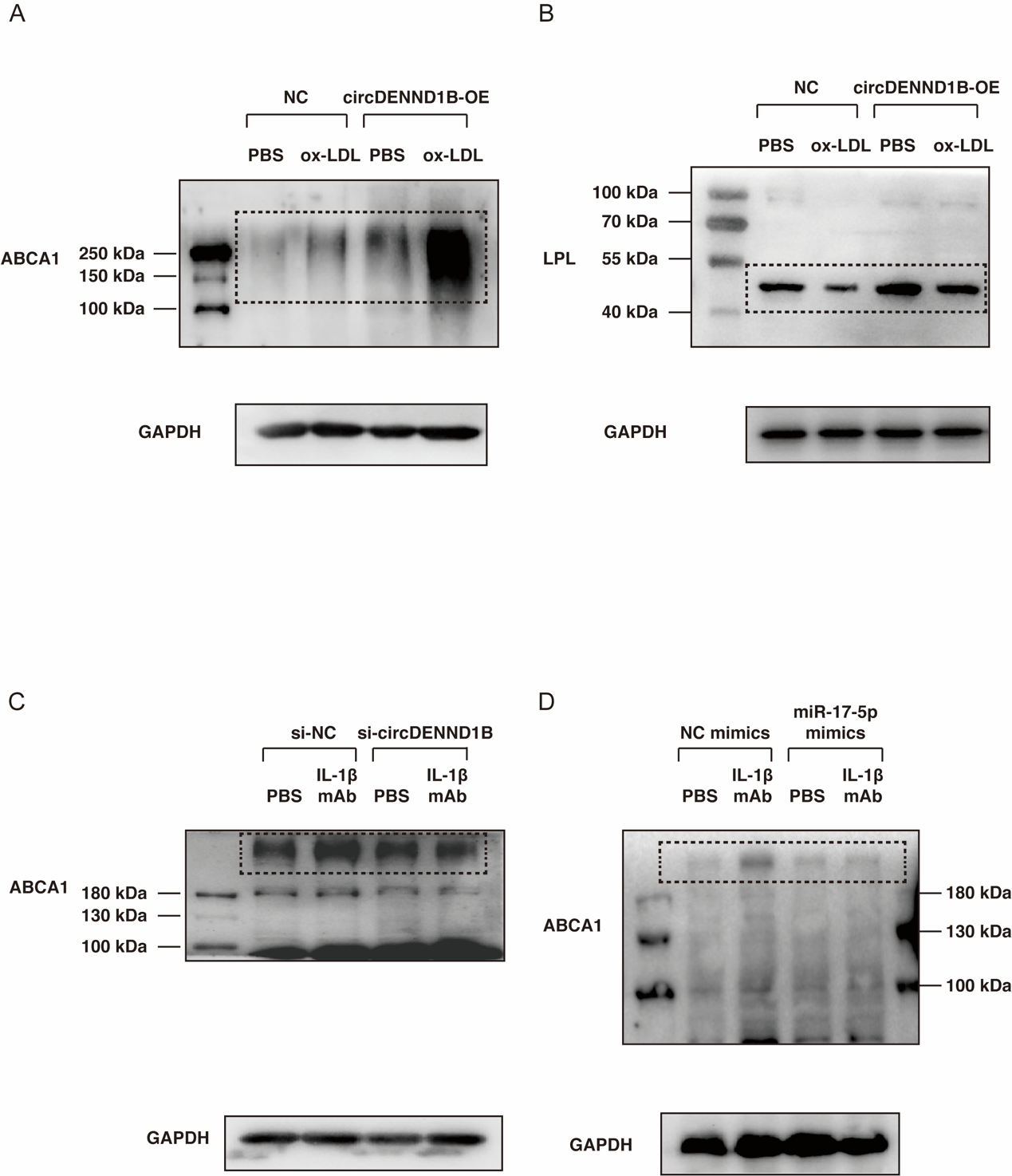


**Figure S7. Uncropped images of WB.** (A-B) The original uncropped blot image of Figure 3J. (C) The original uncropped blot image of Figure 4E. (D) The original uncropped blot image of Figure 5N.
