# Supplementary material for: circDENND1B Participates in the Antiatherosclerotic Effect of IL-1β Monoclonal Antibody in Mouse by Promoting Cholesterol Efflux via miR-17-5p/Abca1 Axis
Source: Front Cell Dev Biol. 2021 Apr 29;9:652032. doi: 10.3389/fcell.2021.652032 (PMC8116881; doi:10.3389/fcell.2021.652032)
Supplement: Supplementary file 2 [file Table_1.docx]

**Table S1. Primers used for quantitative real-time PCR (qRT-PCR) and PCR**

| qRT-PCR primers | | |
| --- | --- | --- |
| Target Name | Forward Primer | Reverse Primer |
| *Il-1β* | TGGACCTTCCAGGATGAGGACA | GTTCATCTCGGAGCCTGTAGTG |
| *Il-6* | TACCACTTCACAAGTCGGAGGC | CTGCAAGTGCATCATCGTTGTTC |
| *Tnf-α* | GGTGCCTATGTCTCAGCCTCTT | GCCATAGAACTGATGAGAGGGAG |
| *Abca1* | GGAGCCTTTGTGGAACTCTTCC | CGCTCTCTTCAGCCACTTTGAG |
| *Abcg1* | GACACCGATGTGAACCCGTTTC | GCATGATGCTGAGGAAGGTCCT |
| *Msr1* | CGCACGTTCAATGACAGCATCC | GCAAACACAAGGAGGTAGAGAGC |
| *Cd36* | GGACATTGAGATTCTTTTCCTCTG | GCAAAGGCATTGGCTGGAAGAAC |
| *Acat1* | GCAGGGAAGTTTGCCAGTGAGA | GAACACGGTCTTGAGCTTTGGC |
| *Lpl* | GCGTAGCAGGAAGTCTGACCAA | AGCGTCATCAGGAGAAAGGCGA |
| *Gapdh* | CATCACTGCCACCCAGAAGACTG | ATGCCAGTGAGCTTCCCGTTCAG |
| circDENND1B  (divergent) | AGCAAATTAAGCACTGAAGTTCTG | GAGCCTGCAGAACCCAAATC |
| circRNA690 | AGGAGATGAAGAGGATGCAT | ATGGCCTCGATCATGTCCAT |
| circRNA994 | TGTGGATAAAAGCTTGGAAGG | AATGCTCGTAGGTGACACAC |
| mmu-circ-0015075 | TGGAGCACTCGGCCATAAAG | ATCAGCATCGTTGACTTGACA |
| circRNA1984 | CTCCAGCAAGAAAGTACATCT | CAACTGCCGAGTCTTGTCTG |
| mmu-let-7b-5p | TGAGGTAGTAGGTTGTGTGGTT | - |
| mmu-miRNA-17-5p | CAAAGTGCTTACAGTGCAGGTAG | - |
| mmu-miRNA-212-5p | ACCTTGGCTCTAGACTGCTTACT | - |
| *mU6* | GCTTCGGCAGCACATATACTAAAAT | - |

**Table S2. Sequences of siRNA and miRNA mimics**

| Target Name | Sense (5’-3’) | Antisense (5’-3’) |
| --- | --- | --- |
| si-NC | UUCUCCGAACGUGUCACGUTT | ACGUGACACGUUCGGAGAATT |
| si-circDENND1B | GAGGCAGAAACACCUGACATT | UGUCAGGUGUUUCUGCCUCTT |
| NC mimics | UUCUCCGAACGUGUCACGUTT | ACGUGACACGUUCGGAGAATT |
| miR-17-5p mimics | CAAAGUGCUUACAGUGCAGGUAG | ACCUGCACUGUAAGCACUUUGUU |
